# Intrauterine infusion of platelet‐rich plasma is a treatment method for patients with intrauterine adhesions after hysteroscopy

**DOI:** 10.1002/ijgo.13353

**Published:** 2020-09-21

**Authors:** Jintao Peng, Manchao Li, Haitao Zeng, Zhi Zeng, Jiana Huang, Xiaoyan Liang

**Affiliations:** ^1^ Reproductive Medicine Research Center the Sixth Affiliated Hospital of Sun Yat‐sen University Guangzhou China

**Keywords:** American Fertility Society score, Balloon, Chemical pregnancy, Hysteroscopy, Intrauterine adhesions, Platelet‐rich plasma

## Abstract

**Objective:**

To evaluate the efficacy of an intrauterine infusion of platelet‐rich plasma (PRP) in patients with intrauterine adhesions (IUAs).

**Methods:**

A retrospective study was conducted from April 2018 to December 2019 to compare the efficacy of intrauterine infusion of PRP with balloon for patients with IUAs. All patients had moderate or severe IUAs, including 28 patients with intrauterine infusion of PRP (group A), 22 patients with intrauterine balloon (group B), and 20 patients with both intrauterine infusion of PRP and balloon in the first operative hysteroscopy. American Fertility Society (AFS) score and rates of chemical pregnancy were compared.

**Results:**

The AFS score decreased with an average of 5.18 ± 3.93, 4.91 ± 4.39, and 5.15 ± 3.17 comparing the third hysteroscopy with the first operative hysteroscopy in group A, group B, and group C, respectively. No significant differences were found among these groups (*P*=0.734). The rates of chemical pregnancy were 40.0% in group A, 38.9% in group B, and 33.3% in group C without significant differences (*P*=0.944).

**Conclusion:**

There were no significant differences between intrauterine infusion of PRP and balloon. PRP is a treatment method for IUAs.

## Abstract 2

There were no significant differences between intrauterine infusion of platelet‐rich plasma and a balloon, and platelet‐rich plasma is a treatment for intrauterine adhesions.

## 1 INTRODUCTION

Intrauterine adhesions (IUAs) are the major cause of uterine infertility and are characterized by endometrial damage because of endometritis or curettage.^1^ Transcervical resection of the adhesions by hysteroscopy is the most effective and commonly used treatment method for IUAs,^1^ followed by hormonal therapy, an intrauterine device or intrauterine balloon, cross‐linked sodium hyaluronate, and oral antibiotics to prevent recurrent IUAs. However, high grades of IUAs mean increased risks of the spontaneous recurrence of adhesions.^2^ For patients with severe IUAs, the incidence of spontaneous recurrent IUAs was reported to be 62.5%.^2^

Platelet‐rich plasma (PRP)—which has the potential to repair tissues including tendons, muscles, cartilage, and ligament—is increasingly used in orthopedics.^3^ Clinical trials and retrospective cohort studies have shown that PRP is considered safe.^3^ PRP decreases fibroblastic activity in animal experiments.^4^ PRP treatment has also been applied in the treatment of hair loss, vulvar lichen sclerosus, lichen planopilaris, and other medical conditions. PRP also plays a positive role in the rejuvenation of tissue and wound healing.^5^ Autologous PRP could promote endometrial growth and improve endometrial regeneration and endometrial capacity. The study by Chang et al.^6^ observed successful endometrial growth after intrauterine infusions of PRP in all five patients who were pregnant. Endometrial repair and the prevention of recurrent IUAs are the key objectives after the hysteroscopic separation of IUAs. The aim of the present study was to evaluate the efficacy of PRP in the treatment of IUAs.

## 2 PATIENTS AND METHODS

### 2.1 Participant criteria

The present study enrolled infertile women with moderate or severe IUAs. Patients with uterine malformations, endometrial polyps, submucous myomas, intrauterine hyperplasia, malignancies of the female reproductive system, premature ovarian failure, and/or endometrial tuberculosis were excluded. The study was approved by the Ethics Committee of the Sixth Affiliated Hospital of Sun Yat‐sen University.

According to the AFS system,^7^ AFS scores of 9–12 were regarded as severe IUAs, while AFS scores of 5–8 were regarded as moderate IUAs, and AFS scores of 1–4 were regarded as slight IUAs. Ninety‐four patients with moderate (61 patients) or severe IUAs (33 patients) were initially included in the retrospective study from April 2018 to December 2019. Among these patients, 38 had intrauterine infusions of PRP (group A), 32 patients had intrauterine balloons (group B; J‐BUS balloon, COOK, Bloomington, MN, USA), and 24 patients had both intrauterine infusions of PRP and intrauterine balloons (group C) in the first operative hysteroscopy.

### 2.2 Preparation of PRP and PRP activator

The PRP was prepared from a modified method from Yamaguchi et al.^8^ On the day of the first hysteroscopy, 15 mL of peripheral venous blood was drawn with a syringe with 5 mL of anticoagulant solution (ACD anticoagulant; Shanghai GENMED Medicine Technology Co., Ltd., Shanghai, China) and then centrifuged at 200 *g* for 10 minutes. Three layers could be found, and the upper and mid layers (plasma layer and buffy coat layer, respectively) were collected into another tube. Then they were centrifuged at 500 g for 10 minutes. The pellet of platelets was mixed with 1 mL of supernatant and 0.5–1 mL of PRP could be obtained.^6^ The concentration of platelets is 3662.0 ± 1351.1 (2SD) × 10^9^/L.

The PRP activator was prepared as follows: 2 mL of 5% calcium chloride injection (Shanghai Xinyi Jinzhu Pharmaceutical Co., Ltd., Shanghai, China) was mixed with 10 mL of 0.9% sodium chloride injection (Otsuka Pharmaceutical Co., Ltd., Tianjin, China) and then centrifuged immediately at 200 g for 1 minute. Then, 2 mL of the mixed solution was discarded and the remaining 10 mL of the mixture was preserved (solution A). Two bottles of Thrombin 500 U (Hunan Yige Pharmaceutical Co., Ltd., Xiangtan, China) were then dissolved with 10 mL of 0.9% sodium chloride injection (Otsuka Pharmaceutical Co., Ltd., Tianjin, China.) (solution B). The PRP activator was the mixture of solution A and solution B, with a ratio of 1:1.

The mixture of PRP and PRP activator (with a ratio of 1:1) would be infused into the uterine cavity with a catheter for intrauterine insemination (Shenzhen Huanhao Technologies Co., Ltd., Shenzhen, China).

### 2.3 Routine procedure and assessment of treatment

Each patient signed an informed consent form before surgery. In the first operative hysteroscopy (Storz, Tuttlingen, Germany), intrauterine infusion of PRP, intrauterine balloon, and both intrauterine infusion of PRP and intrauterine balloon were applied in group A, group B, and group C, respectively. The routine included a second‐look hysteroscopy that was carried out 1 week after the first operation and a third‐look hysteroscopy that was carried out in the follicular phase of the next menstrual cycle to investigate whether there were postoperative adhesions. Hormone replacement therapy (HRT) was applied after the first operation. Blunt dissection was applied if there were recurrent IUAs in the second‐look hysteroscopy. Surgical complications were recorded, including uterine perforation, anesthesia accidents, and infection after the operation. After the routine treatment, the transfer of embryos would be performed if the patients had embryos in the study center and serum β‐HCG would be tested 12 or 14 days after the transfer (blastocyst‐stage embryo or cleavage‐stage embryo, respectively). The primary outcome was the change of AFS score and the secondary outcome was the rate of chemical pregnancy. Statistical comparisons of the basic characteristics of included patients and the change of AFS scores were performed using a non‐parametric statistical test. Statistical comparison of the rate of chemical pregnancy was performed using a χ^2^ test. The tests were performed using Statistical Program for Social Sciences (SPSS 25.0, IBM, Armonk, NY, USA). *P*<0.05 was considered statistically significant.

## 3 RESULTS

In the present study, 38 patients in group A, 32 patients in group B, and 24 patients in group C were initially included. Some patients did not undergo the third‐look hysteroscopy for personal reasons so only 28 patients in group A, 22 patients in group B, and 20 patients in group C were included in the final analysis. None of the patients in the final analysis received blunt dissection in the second‐look hysteroscopy because no recurrent IUAs were found among them 1 week after the first operative hysteroscopy. No surgical complications were found. Analyzing the AFS scores in the third‐look hysteroscopy, six patients in group A had an AFS score of 5, five patients and one patient in group B had AFS scores of 5 and 8, respectively, and seven patients in group C had an AFS score of 5. Table 1 summarizes the basic characteristics of the participants in the final analysis.

**Table 1:** Basic characteristics of the participants in groups A, B, and C in the final analysis.

Group	Number of patients	Age (years)		Artificial abortion or curettage		Hypomenorrhea	History of hysteroscopic surgery with IUA	
		Mean	2SD	Mean	2SD		Mean	2SD
A	28	35.07	8.55	1.32	2.39	28	0.46	1.25
B	22	34.45	11.98	1.32	1.94	22	0.32	1.11
C	20	33.90	10.75	1.30	2.11	20	0.55	1.17
*P*	—	0.835		0.978		—	0.367	

The mean AFS scores decreased from 7.93 ± 3.20 in the first operative hysteroscopy to 2.75 ± 2.81 in the third hysteroscopy in group A. The mean AFS scores decreased from 8.09 ± 3.71 in the first operative hysteroscopy to 3.18 ± 3.49 in the third hysteroscopy in group B, and they decreased from 8.20 ± 3.26 to 3.05 ± 3.25 in group C. The AFS score decreased with an average of 5.18 ± 3.93, 4.91 ± 4.39, and 5.15 ± 3.17, comparing the third hysteroscopy with the first operative hysteroscopy in group A, group B and group C, respectively. No significant differences were found among these groups when comparing the change in AFS score (*P*=0.734). The rates of chemical pregnancy were 40.0% in group A (six patients with positive serum β‐HCG among 15 patients with transfer of embryos), 38.9% in group B (seven patients with positive serum β‐HCG among 18 patients with transfer of embryos), and 33.3% in group C (three patients with positive serum β‐HCG among nine patients with transfer of embryos) (*P*=0.944).

## 4 DISCUSSION

The presence of IUAs impacts subsequent pregnancies^9^ and hysteroscopic procedures are the most common treatment for patients with IUAs. Despite a successful initial surgery, the reformation of IUAs occurred in approximately one‐third of patients and the incidence of recurrent IUAs was reported to be approximately two‐thirds of women with severe IUAs.^2^ Endometrium growth is one of the most important factors in pregnancy, so the prevention of recurrent IUAs and promotion of endometrial repair are the key objectives that should be considered after hysteroscopic surgery. Barrier gels, hormonal treatment, and intrauterine balloons are usually applied in patients with IUAs after an operative hysteroscopy.^10^ However, no significant or clear improvements in clinical symptoms and rates of subsequent pregnancies have been found.^11^, ^12^ In 2006, Amer and Abd‐El‐Maeboud^13^ found that an amniotic membrane was an effective method for reducing the recurrence of IUAs and promoting endometrial regeneration. However, amniotic membranes did not affect the incidence of recurrence of IUAs or the rate of pregnancy.^14^ For patients with recurrent IUAs, allogeneic cell therapy with mesenchymal stem cells (MSCs) from the umbilical cord was a safe and effective therapeutic method.^15^

PRP is defined as the plasma fraction of autologous blood with platelet numbers that are enhanced four‐ to six‐fold compared with that of whole blood.^16^ There was no significant difference in the change of AFS scores among these three groups in the present study. Furthermore, there was no significant difference in rates of chemical pregnancy after embryo transfers among these groups. PRP can play an important role in wound healing, preventing recurrent IUAs, and promoting endometrial repair. First, the fibrin and high concentrations of platelets in the PRP contribute to hemostasis and prevent acute blood loss after hysteroscopic surgery.^17^, ^18^ The fibrin and platelets can promote wound healing after hysteroscopy. Second, white blood cells in the PRP may play an important role against infection after hysteroscopy. Third, unlike the intrauterine balloon, the fluidity of PRP helps the patient to avoid distending pain and foreign body sensations. In addition, PRP is not expensive. Fourth, PRP is autologous; therefore, it can avoid transplant rejection. The risk of transmission of disease and immunogenic reactions can also be avoided.^19^ Finally, and most importantly, growth factors, including TGF‐β, PDGF, EGF‐F, VEGF, and FGF, can promote the healing process.^18^, ^19^ Growth factors can also regulate cell migration, attachment, and proliferation and promote the accumulation of extracellular matrix.^20^ Moreover, growth factors can stimulate neovascularization of the endometrium and promote endometrial repair. Angiogenesis promoted by growth factors plays a critical role in tissue growth and repair.^21^ The estrogen in hormone treatments contributes to thrombosis and fibrosis, which may result in recurrent IUAs or unsatisfactory endometrial repair.^22^ This may be resolved by the growth factors in PRP.

Early second‐look hysteroscopic examinations within 2 months may improve clinical outcomes^23^, ^24^; therefore, an advanced second‐look hysteroscopy was carried out after the first operative hysteroscopy, and a third‐look hysteroscopy was carried out in the follicular phase of the next menstrual cycle as part of routine treatment. No significant recurrent IUAs were found in the second‐look hysteroscopy so none of the patients included in the final analysis received blunt dissection in the second‐look hysteroscopy. Satisfactory results were achieved in the cases with a third‐look hysteroscopy, including results indicating endometrial repair and a normal—or almost normal—uterine cavity. Significantly decreased AFS scores were found in all groups with a third‐look hysteroscopy. No surgical complications were observed in the three groups, and the intrauterine infusion of PRP is considered safe, as reported in other studies.^25^

In conclusion, there were no significant differences between the intrauterine infusion of PRP and the intrauterine balloon. PRP is a form of treatment for IUAs after operative hysteroscopy and may be a substitute for the intrauterine balloon. However, randomized controlled trials with large sample sizes are warranted to further confirm the conclusions of the present study and to compare the efficacy of intrauterine infusions of PRP with intrauterine balloons applied immediately after transcervical resection of the adhesions by hysteroscopy in patients with IUAs.

## AUTHOR CONTRIBUTIONS

JP and ML contributed to data collection, data analysis, and writing the manuscript. Both JP and ML contributed equally to this study. XL contributed to the study design and revising the manuscript. JP, ML, HZ, and ZZ performed the surgical procedures. JH contributed to data collection. All authors approved the final version of the manuscript for publication.

## CONFLICTS OF INTEREST

The authors have no conflicts of interest.
